# Two Sides of a Coin: a Zika Virus Mutation Selected in Pregnant Rhesus Macaques Promotes Fetal Infection in Mice but at a Cost of Reduced Fitness in Nonpregnant Macaques and Diminished Transmissibility by Vectors

**DOI:** 10.1128/JVI.01605-20

**Published:** 2020-11-23

**Authors:** Danilo Lemos, Jackson B. Stuart, William Louie, Anil Singapuri, Ana L. Ramírez, Jennifer Watanabe, Jodie Usachenko, Rebekah I. Keesler, Claudia Sanchez-San Martin, Tony Li, Calla Martyn, Glenn Oliveira, Sharada Saraf, Nathan D. Grubaugh, Kristian G. Andersen, James Thissen, Jonathan Allen, Monica Borucki, Konstantin A. Tsetsarkin, Alexander G. Pletnev, Charles Y. Chiu, Koen K. A. Van Rompay, Lark L. Coffey

**Affiliations:** a University of California, Davis, School of Veterinary Medicine, Department of Pathology, Microbiology and Immunology, Davis, California, USA; b University of California, Davis, California National Primate Research Center, Davis, California, USA; c University of California, San Francisco, Department of Laboratory Medicine, San Francisco, California, USA; d The Scripps Research Institute, San Diego, California, USA; e Department of Epidemiology of Microbial Diseases, Yale School of Public Health, New Haven, Connecticut, USA; f Lawrence Livermore National Laboratory, Livermore, California, USA; g Laboratory of Infectious Diseases, National Institute of Allergy and Infectious Diseases, National Institutes of Health, Bethesda, Maryland, USA; Cornell University

**Keywords:** Zika virus, arbovirus, congenital Zika syndrome, emergence, experimental infection, fitness, flavivirus, mouse, mutation, nonhuman primate

## Abstract

Although fetal death is now understood to be a severe outcome of congenital Zika syndrome, the role of viral genetics is still unclear. We sequenced Zika virus (ZIKV) from a rhesus macaque fetus that died after inoculation and identified a single intrahost substitution, M1404I, in the ZIKV polyprotein, located in nonstructural protein 2B (NS2B). Targeted sequencing flanking position 1404 in 9 additional macaque mothers and their fetuses identified M1404I at a subconsensus frequency in the majority (5 of 9, 56%) of animals and some of their fetuses. Despite its repeated presence in pregnant macaques, M1404I has occurred rarely in humans since 2015. Since the primary ZIKV transmission cycle is human-mosquito-human, mutations in one host must be retained in the alternate host to be perpetuated. We hypothesized that ZIKV I1404 increases viral fitness in nonpregnant macaques and pregnant mice but is less efficiently transmitted by vectors, explaining its low frequency in humans during outbreaks. By examining competitive fitness relative to that of ZIKV M1404, we observed that ZIKV I1404 produced lower viremias in nonpregnant macaques and was a weaker competitor in tissues. In pregnant wild-type mice, ZIKV I1404 increased the magnitude and rate of placental infection and conferred fetal infection, in contrast to ZIKV M1404, which was not detected in fetuses. Although infection and dissemination rates were not different, Aedes aegypti mosquitoes transmitted ZIKV I1404 more poorly than ZIKV M1404. Our data highlight the complexity of arbovirus mutation-fitness dynamics and suggest that intrahost ZIKV mutations capable of augmenting fitness in pregnant vertebrates may not necessarily spread efficiently via mosquitoes during epidemics.

**IMPORTANCE** Although Zika virus infection of pregnant women can result in congenital Zika syndrome, the factors that cause the syndrome in some but not all infected mothers are still unclear. We identified a mutation that was present in some ZIKV genomes in experimentally inoculated pregnant rhesus macaques and their fetuses. Although we did not find an association between the presence of the mutation and fetal death, we performed additional studies with ZIKV with the mutation in nonpregnant macaques, pregnant mice, and mosquitoes. We observed that the mutation increased the ability of the virus to infect mouse fetuses but decreased its capacity to produce high levels of virus in the blood of nonpregnant macaques and to be transmitted by mosquitoes. This study shows that mutations in mosquito-borne viruses like ZIKV that increase fitness in pregnant vertebrates may not spread in outbreaks when they compromise transmission via mosquitoes and fitness in nonpregnant hosts.

## Abstract (precis)

Although Zika virus infection of pregnant women can result in congenital Zika syndrome, the factors that cause the syndrome in some but not all infected mothers are still unclear. We identified a mutation that was present in some ZIKV genomes in experimentally inoculated pregnant rhesus macaques and their fetuses. Although we did not find an association between the presence of the mutation and fetal death, we performed additional studies with ZIKV with the mutation in nonpregnant macaques, pregnant mice, and mosquitoes. We observed that the mutation increased the ability of the virus to infect mouse fetuses but decreased its capacity to produce high levels of virus in the blood of nonpregnant macaques and to be transmitted by mosquitoes. This study shows that mutations in mosquito-borne viruses like ZIKV that increase fitness in pregnant vertebrates may not spread in outbreaks when they compromise transmission via mosquitoes and fitness in nonpregnant hosts.

## INTRODUCTION

Congenital Zika syndrome (CZS), caused by Zika virus (ZIKV), produces a disease spectrum that sometimes results in microcephaly or death in fetuses from mothers infected during pregnancy. In North and South American outbreaks since 2015, about 15% of fetuses from ZIKV-infected mothers displayed reduced growth, sensory disorders, and central nervous system malformations (1–6), manifestations of CZS (7–9). CZS abnormalities are associated with the detection of ZIKV RNA or infectious virus in amniotic fluid (AF) and fetal tissues, including brain (4–6, 10–14). Viral and host factors that affect the severity of CZS, including why some fetuses die or develop microcephaly while others do not, are still not well understood.

Mutations in ZIKV that arise and spread in humans during outbreaks may contribute to CZS or modify transmission by mosquitoes. A substitution in the nonstructural protein 1 (NS1) increases infectivity in Aedes aegypti vectors (15). However, identifying mutations that may influence the ZIKV phenotype is complicated, as consensus (average nucleotide) genomes from febrile human cases in recent outbreaks differ by hundreds of nonsynonymous mutations compared to the sequences of ancestral genomes (16). Any of these mutations alone or in combination could modify incidence, transmissibility, or pathogenesis. Furthermore, the consensus genomes from 1 miscarriage and 7 microcephaly cases were found to be interleaved with genomes from febrile cases in phylogenies and shared no common amino acid differences from the genomes from febrile patients living in the same areas, suggesting that no single mutation or group of mutations associates with the most severe CZS outcomes (17). Phylogenetic inference identified a prM protein-coding substitution (prM-S139N) that increased neurovirulence in mice inoculated intracranially (18), although this finding was not reproducible in repeated studies (19). Another substitution identified via phylogenetic analysis, E-V473M, increased viremias in nonpregnant cynomolgus macaques and neurovirulence in 1-day-old mice inoculated intracranially but did not modify ZIKV RNA levels in A. aegypti mosquitoes (20). Since prM-S139N and E-V473M have been found to be present in most genomes from North and South American outbreaks since 2015, including in febrile women whose babies did not develop CZS, they are likely not major determinants of the CZS outcome in pregnant women.

Intrahost ZIKV evolution has been less well studied than evolution across patients but may also affect disease outcome. Defining intrahost evolution over time necessitates repeated sampling. For pregnant women, sequencing of ZIKV from even a single point in time is often unsuccessful, since viremia is frequently missed in the clinical setting (14, 21), and AF is rarely available, given that amniocentesis can lead to iatrogenic infection (22) or ZIKV transmission to the fetus from an infected mother. Due to these limitations, the role of intrahost viral genetics in CZS remains unclear. To circumvent limited human sample availability or the testing of hundreds of mutations identified via phylogenetics as potential determinants of CZS, we used an experimental model to identify intrahost ZIKV mutations in infected pregnant rhesus macaques with known CZS outcomes.

Macaques have rapidly become an important model for understanding ZIKV infection and disease (23–35), due to placentation, immunology, fetal organogenesis, and neurologic development in macaques similar to humans. Studies using rhesus macaques, including our own work (25, 35), have demonstrated fetal central nervous system lesions consistent with the abnormal brain development observed in CZS. As in humans, not all rhesus macaque fetuses from mothers inoculated with the same ZIKV stock at similar gestational windows develop CZS. While host determinants certainly play a role, intrahost viral mutations that develop may also influence different CZS outcomes. Mutants with subconsensus mutations in genetically heterogeneous populations of many other RNA viruses (36–51) have been associated with modified transmissibility or disease outcomes. A wild-type (WT) 2015 ZIKV strain from a febrile patient in Brazil showed an augmented replicative success in human placental and neural cells compared to its genetically homogeneous infectious clone derivative (17). This highlights that intrahost ZIKV mutants play a role in infection kinetics, even in cell monocultures. However, subconsensus ZIKV mutations in an individual host are unrecognized without deep sequencing, which characterizes the entire population of viral RNA genomes instead of solely the consensus. Intrahost ZIKV populations from 11 pregnant rhesus macaques revealed no *de novo* mutations in one study (51), although sequencing of ZIKV from maternal serum and AF was done at just one time point postinoculation.

In this study, we sequenced intrahost ZIKV from a rhesus macaque mother and her deceased fetus and identified a single subconsensus mutation, M1404I, in the NS2B coding region. We then focused sequencing at position 1404 in ZIKV from 9 additional pregnant rhesus macaques and their fetuses, where we found that it was present at a minority frequency in 5 additional animals but was not detectable or was present at a frequency of ∼1% in the inoculum. Given its rise in frequency, we hypothesized that the mutant confers increased fitness in rhesus macaques and, by extension, other vertebrates. We therefore performed parallel infections with ZIKV M1404 and ZIKV I1404 in nonpregnant rhesus macaques and pregnant mice as well as mixed infections in nonpregnant rhesus macaques. Since ZIKV is primarily mosquito borne, viral genomes that evolve in vertebrates must be maintained in mosquitoes to persist in alternating vertebrate-mosquito-vertebrate cycling. To test this concept and to better understand why ZIKV I1404 was detected at a low frequency in humans during outbreaks, despite increasing in frequency intrahost in pregnant rhesus macaques, we compared the transmissibility of ZIKV M1404 and ZIKV I1404 by parallel infections of Aedes aegypti vectors.

## RESULTS

### A ZIKV mutation arises *de novo* or increases in frequency in experimentally inoculated pregnant rhesus macaques.

**FIG 1:**
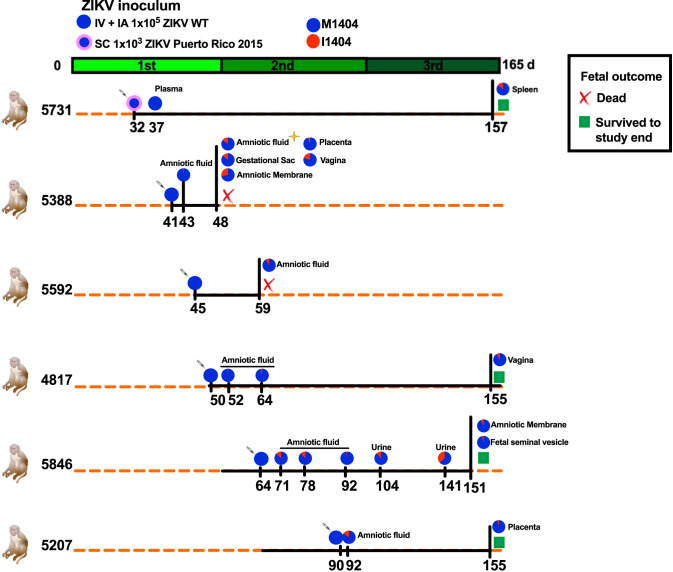
A ZIKV mutant arose or increased in frequency in six pregnant rhesus macaques inoculated with two different ZIKV strains via two routes. Experimental design for inoculation of pregnant rhesus macaques with 1 × 10^5^ ZIKV WT (a 2015 Brazilian strain [GenBank accession number KU321639.1]) both intravenously (i.v.) and intra-amniotically (i.a.) (blue circle) or 1 × 10^3^ of ZIKV Puerto Rico 2015 (GenBank accession number KU501215.1) delivered subcutaneously (s.c.) (blue circle with a thick pink border) on the days indicated by syringes. For more details on the study design, see the work of Coffey et al. (25) and Van Rompay et al. (35). Both inocula contained mostly ZIKV M1404, where ZIKV I1404 was absent or present at the limit of detection, ∼1%. The green line shows the duration of each rhesus macaque pregnancy divided into 3 ∼55-day trimesters, where full term is 165 days. The orange dotted lines represent 165 days of gestation, and the black solid lines show the period of infection and the experiment end for each dam and its fetus. Fetal death is shown as a red X. Fetuses that survived to the study endpoint, gestation day 155, are indicated with green squares. The gold star represents the time of first detection of I1404; full genome sequencing of this specimen showed no other genome-wide mutations. The pie charts represent the relative abundance of the amino acid at position 1404. The I1404 mutation was not detected in 4 additional pregnant macaques in the same study (not shown here but included in Table S2 in the supplemental material). Table S2 shows the depth of sequencing coverage for the data represented in the pie charts. The tissues listed are maternal unless otherwise indicated.

Pregnant rhesus macaques were inoculated with ZIKV to study viral infection dynamics and CZS. Outcomes from those experiments are detailed in the work of Coffey et al. (25) and Van Rompay et al. (35). The rhesus macaques in those studies were inoculated in the first or second trimester of their pregnancy intravenously (i.v.) and intra-amniotically (i.a.) with 1 × 10^5^ PFU of the ZIKV WT (strain SPH2015, a Brazilian strain [GenBank accession number KU321639]) or subcutaneously (s.c.) with 1 × 10^3^ PFU of ZIKV Puerto Rico 2015 (strain PRVABC59 [GenBank accession number KU501215]) (Fig. 1). Some fetuses died preterm, while most survived to the end of the study, which was ∼10 days preterm. To investigate intrahost dynamics, we analyzed samples archived from those rhesus macaques by deep sequencing. We first sequenced the complete ZIKV genome in amniotic fluid (AF) from rhesus macaque 5388, whose fetus died at 7 days postinoculation (dpi); multiple maternal and fetal tissues exhibited high ZIKV RNA levels (23). We identified no consensus mutations (mutations found in >50% of RNA reads) and only one subconsensus mutation (mutations found in <50% of RNA reads), G4315A, which occurred at an 18% frequency. The G4315A mutation results in a nonsynonymous methionine (M)-to-isoleucine (I) substitution at amino acid position 1404 of the ZIKV polyprotein (with reference to the sequence of WT strain SPH2015 [GenBank accession number KU321639]) and is located in the nonstructural protein 2B (NS2B), a cofactor for the flavivirus protease, NS3. Sequencing of the inoculum flanking the G4315A variant locus by 2 different laboratories at 5,705- and 2,296-fold coverage revealed the mutant in 0.2% or 1.3% of the ZIKV RNA reads, respectively (see Table S2 in the supplemental material), at or below the reported error rates for short-read Illumina sequencing (52). We performed additional sequencing to determine whether ZIKV M1404I was also present in other maternal or fetal tissues from rhesus macaque 5338 and also in 9 additional pregnant ZIKV-inoculated rhesus macaques with different CZS outcomes using a targeted sequencing approach flanking position 1404. In addition to the AF, where it was first detected, ZIKV M1404I was detected in the gestational sac, amniotic membrane, placenta, and vagina of macaque 5388. ZIKV M1404I was also detected at a minority frequency in 4 additional pregnant rhesus macaques inoculated i.v. and i.a. with the WT Brazilian strain in multiple tissues, including the amniotic fluid, gestational sac, amniotic membrane, placenta, maternal vagina, fetal seminal vesicle, and maternal urine. The M1404I mutation was also detected in the spleen of one mother (macaque 5731) that had been inoculated s.c. with the isolate from Puerto Rico. The Puerto Rican ZIKV inoculum lacked the I1404 variant in any RNA reads sequenced at a coverage depth of 2,242-fold. The I1404 mutation was not detected in tissues from 4 additional pregnant rhesus macaques in the same studies (Table S2). Although the mutation was detected in 2 rhesus macaques whose fetuses died, it was also found in 4 rhesus macaques whose fetuses survived to near term, indicating that it was not associated with fetal death. The *de novo* development of ZIKV M1404I and its increase in frequency from its near absence in the inoculum to its presence within multiple tissues intrahost in six pregnant rhesus macaques inoculated by two routes with two different ZIKV strains suggested that M1404I may be a rhesus macaque-adaptive substitution. To study the mutation in isolation, we generated infectious clone-derived viruses that varied only at position 1404 for comparative fitness experiments in cells, nonpregnant rhesus macaques, pregnant mice, and mosquitoes.

### The growth kinetics of ZIKV I1404 are superior to those of ZIKV M1404 in vertebrate cells.

**FIG 2:**
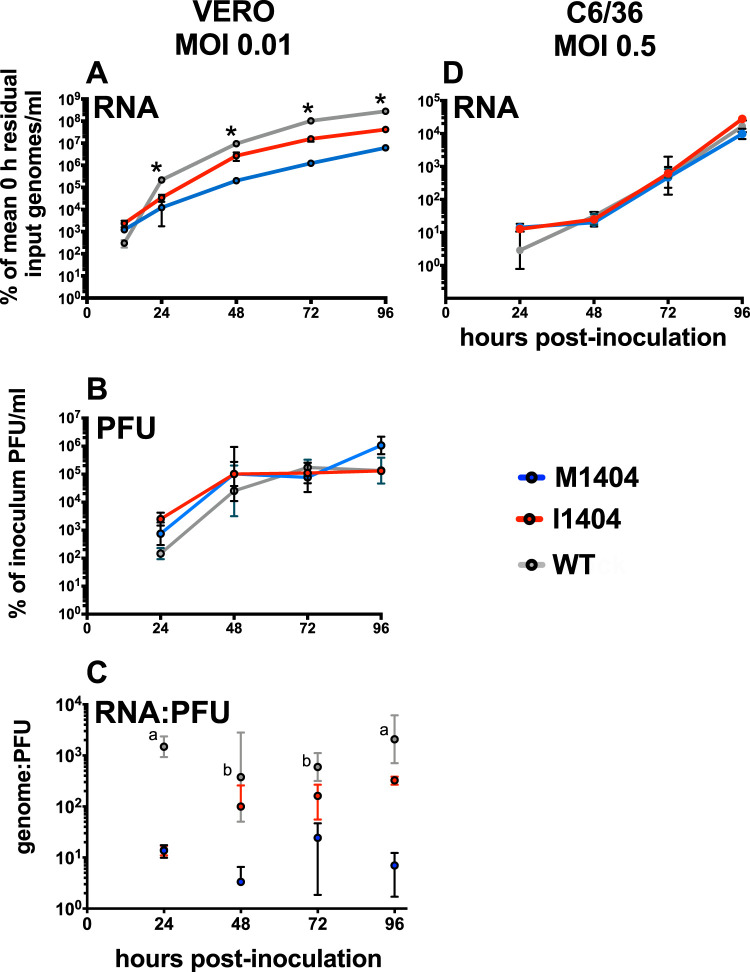
Growth kinetics of ZIKV M1404, ZIKV I1404, and the WT in African green monkey kidney (Vero) and Aedes albopictus (C6/36) cells. (A) ZIKV I1404 exhibits ZIKV RNA growth kinetics superior to those of ZIKV M1404 from 24 to 96 hpi in Vero cells at a multiplicity of infection (MOI) of 0.01 PFU/cell. *, *P* < 0.001 across all groups. (B) Infectious ZIKV levels in Vero cells from 24 to 96 hpi are not different between ZIKV M1404, ZIKV I1404, and the WT (*P* > 0.05). (C) Genome/PFU ratios for WT ZIKV are higher than those for ZIKV M1404 from 24 to 96 hpi and at 24 and 96 hpi compared to those for ZIKV I1404. ^a^, the value for the WT differs from that for both ZIKV M1404 and ZIKV I1404; ^b^, the value for the WT differs from that for ZIKV M1404 only (*P* < 0.05). (D) ZIKV RNA levels in C6/36 cells at an MOI of 0.5 PFU/cell are not different across groups (*P* > 0.05). Each dot for panels A, B, and D represents the mean for 3 replicate supernatant samples, where each supernatant was measured in 3 qRT-PCR replicates. The RNA and PFU measurements for panels A and B were from the same supernatants. The statistical test used was a repeated-measures one-way ANOVA. Error bars show standard deviations for panels A, B, and D, and dots show the geometric mean and geometric standard deviation for panel C.

We tested whether the expression of ZIKV I1404 in cell culture modifies ZIKV growth kinetics relative to those of ZIKV M1404. We generated two infectious clones identical in sequence except for the amino acid at position 1404. To do this, we modified a ZIKV infectious clone made from a 2015 Brazilian ZIKV isolate, Paraiba_01/2015 (17), to match the amino acid sequence of Brazilian strain SPH2015. Strain SPH2015, hereafter termed the WT, was isolated from a patient and passaged in cells before use in pregnant rhesus macaque studies. The sequences of clone-derived ZIKV, termed ZIKV M1404 and ZIKV I1404, based on the amino acid at polyprotein position 1404, were verified by Sanger sequencing (data not shown), using primers flanking the ZIKV genome (Table S1). The relative growth kinetics, measured as ZIKV RNA and infectious virus levels in the supernatants of inoculated cultures over time, were assessed in African green monkey kidney (Vero) or Aedes albopictus larval (C6/36) cells (Fig. 2). ZIKV I1404 exhibited significantly higher ZIKV RNA levels than ZIKV M1404 from 24 to 96 h postinoculation (hpi) in Vero cells at a multiplicity of infection (MOI) of 0.01 PFU/cell (*P* < 0.001, repeated-measures analysis of variance [ANOVA]) (Fig. 2A). ZIKV RNA levels for both ZIKV M1404 and ZIKV I1404 were lower than those for the WT at all times studied, a pattern common to clone-derived ZIKV compared to WT progenitor viruses (16). Even though the increase in ZIKV RNA kinetics was accelerated for the I1404 clone compared to the M1404 clone, infectious ZIKV levels in Vero cells from 24 to 96 hpi by plaque assay were not different between ZIKV M1404, ZIKV I1404, and the WT (*P* > 0.05, repeated-measures ANOVA) (Fig. 2B). While the RNA/PFU ratio was not different between the clones at 24 hpi, the RNA/PFU ratio in Vero cells (Fig. 2C) was higher for the I1404 clone than for the M1404 clone from 48 to 96 hpi, although the I1404 clone was not as high as the WT at 96 hpi (*P* < 0.05, repeated-measures ANOVA). In C6/36 mosquito cells, ZIKV RNA levels after inoculation at an MOI of 0.5 PFU/cell were not different across groups at any time from 24 to 96 hpi (*P* > 0.05, repeated-measures ANOVA) (Fig. 2D). These results indicate that in a standard vertebrate cell line, the I1404 substitution detected in pregnant rhesus macaques increases ZIKV RNA kinetics compared to the kinetics seen with the progenitor M1404 sequence, although it does not change the levels of infectious virus.

### ZIKV I1404 displays reduced fitness in nonpregnant rhesus macaques inoculated with an equal mixture of the M1404 and I1404 clones.

**FIG 3:**
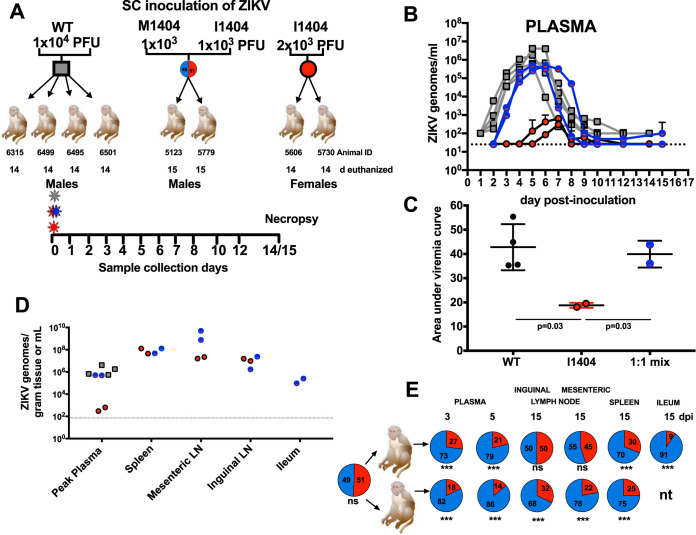
A ZIKV mutant, ZIKV I1404, generates lower viremias and is less abundant than ZIKV M1404 in the tissues of nonpregnant rhesus macaques after mixed inoculation. (A) Experimental design for subcutaneous (s.c.) inoculation of male and nonpregnant female rhesus macaques with the ZIKV WT (left), a 1:1 mixture of infectious clone-derived ZIKV I1404 and ZIKV M1404 (middle), or infectious clone-derived ZIKV I1404 (right) at the indicated doses. The 1:1 mixture was verified by sequencing the inoculum prior to administration to macaques. Blood was collected daily from 1 to 8 dpi, at 10 and 12 dpi, and at either 14 or 15 dpi, at which point the animals were euthanized for tissue collection. d, day. (B) Plasma viremia kinetics for individual rhesus macaques inoculated with the ZIKV WT (gray), the 1:1 mixture (blue), or ZIKV I1404 (red). The dotted line shows the limit of detection, 1.4 × 10^1^ genome copies/ml plasma. Error bars show standard deviations for triplicate measurements. (C) The area under the curve (AUC) for rhesus macaques infected with ZIKV I1404 compared to the WT or the 1:1 mix. AUC statistics were performed on log-transformed viremia measurements (one-way ANOVA). (D) ZIKV RNA levels in tissues of rhesus macaques. LN, lymph node. The dotted line represents the mean limit of detection, 1.9 × 10^1^ ZIKV genome copies/gram tissue or genome copies/ml. (E) Targeted quantitative sequencing of the region flanking ZIKV position 1404, showing the relative abundance of ZIKV M1404 (blue) orZIKV I1404 (red) in the indicated tissues from male rhesus macaques inoculated with the 1:1 mixture (***, *P* < 0.01; ns, not significantly different at *P* = 0.05; *P* values were determined by chi-square tests). nt, not tested. Table S3 in the supplemental material shows the depth of sequencing coverage for the data represented here. Each dot in panels B, C, and D shows the mean for triplicate qRT-PCR ZIKV RNA measurements.

To directly compare the relative fitness of ZIKV M1404 with that of ZIKV I1404, we performed a competition experiment wherein both viruses were inoculated together at an equal ratio into two nonpregnant rhesus macaques (Fig. 3A). Equal ratios were confirmed by titrating the infectivity and measuring the viral RNA levels of both competitors before mixing and then deep sequencing the mixture before inoculation into macaques. Rhesus macaques were also inoculated with the ZIKV WT or ZIKV I1404 alone for comparison. ZIKV M1404 was not included since its coding amino acid sequence is identical to that of the WT, to reduce the number of macaques used. We defined fitness as the plasma viremia magnitude and kinetics and, for mixed infections, the relative abundance of ZIKV RNA reads encoding M1404 or I1404 in plasma and tissues. Rhesus macaques inoculated with the ZIKV WT or the 1:1 mixture of ZIKV M1404 and ZIKV I1404 exhibited similar kinetics (*P* = 0.474, unpaired *t* test), with peak viremias at 5 to 7 dpi that reached 10^6^ to 10^7^ ZIKV genomes/ml of plasma (Fig. 3B). Although the peak viral RNA levels in animals inoculated with the mixture appeared to be lower than those in WT-infected macaques, likely reflecting the reduced fitness of clone-derived flaviviruses compared to the fitness of their WT progenitors, there was no difference in the viremia area under the curve (AUC) (Fig. 3C). In contrast, rhesus macaques inoculated with ZIKV I1404 developed significantly lower peak viremias, 10^2^ ZIKV genomes/ml plasma, that endured for a significantly shorter period of time and showed a lower AUC (*P* = 0.03 for AUC) compared to the other two groups. Despite a reduced magnitude and kinetics of viremia, most tissue ZIKV levels were similar in animals infected with ZIKV I1404, the ZIKV WT, or the 1:1 mixture (Fig. 3D). The I1404 mutation did not revert to M1404 in the spleen or mesenteric lymph node of either ZIKV I1404-inoculated animal at levels detectable by Sanger sequencing (data not shown). Targeted deep sequencing flanking the 1404 locus in rhesus macaques inoculated with the 1:1 mixture detected ZIKV I1404 at a frequency significantly lower than that of ZIKV M1404 in plasma, spleen, ileum, and mesenteric and inguinal lymph nodes (*P* < 0.001, chi-square tests) (Fig. 3E; Table S3). Despite the repeated detection of ZIKV I1404 in pregnant rhesus macaques, these experiments show that I1404 reduces viral fitness relative to M1404 in nonpregnant rhesus macaques. Given that pregnant rhesus macaques studies are labor intensive and costly, we therefore explored whether the fitness advantages conferred by I1404 may be specific to pregnancy using pregnant mice.

### ZIKV I1404 confers fetal infection in pregnant CD-1 mice.

**FIG 4:**
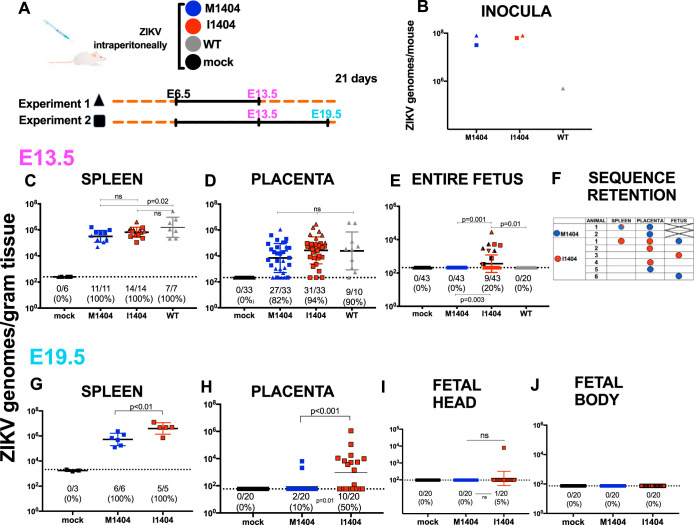
ZIKV I1404 produces higher spleen and placental ZIKV RNA titers and confers fetal infection in infected pregnant mice. (A) Experimental design showing intraperitoneal inoculation of pregnant CD-1 mice with ZIKV M1404 (blue), ZIKV I1404 (red), or the WT (gray) or mock inoculation (DMEM; black) on embryonic day 6 (E6.5) of pregnancy, where full term in mice is E21. Two experiments were performed. In experiment 1 (triangles), dams were euthanized on E13.5. In experiment 2 (squares), dams were euthanized on E13.5 or E19.5. (B) Back titration of residual inocula. (C to E) ZIKV RNA levels in maternal spleens (C), placentas (D), and fetuses (E) at E13.5. The different patterns of shading for the triangles and squares in panel E show the different dams (*n* = 7) from which infected fetuses were detected. (F) Sanger sequencing of maternal tissues or fetuses shows that I1404 reverts to M1404 in some animals. X, no fetuses had detectable ZIKV RNA, so sequencing could not be performed. An empty field indicates that sequencing was not attempted from that sample. (G to J) ZIKV RNA levels in maternal spleens (G), placentas (H), fetal heads (I), and fetal bodies (J) at E19.5. Each dot represents one tissue sample and is reported as the mean from 3 ZIKV RNA qRT-PCR replicates. Group means are shown as black lines and include the results for samples with no detectable ZIKV RNA, which were reported at the limit of detection (LOD). Only the results for samples with 3/3 qRT-PCR replicates with detectable ZIKV RNA are reported above the LOD. The dotted lines denote the LOD, which was a mean of 1.8 × 10^1^ ZIKV RNA copies/gram of tissue for all panels except panel G, where the LOD was 3.3 × 10^3^ ZIKV RNA copies/gram of tissue. Statistical analyses comparing the means used ANOVA multiple comparisons. The rates of ZIKV RNA-positive samples in each group were compared by the use of Fisher’s exact statistics.

**FIG 5:**
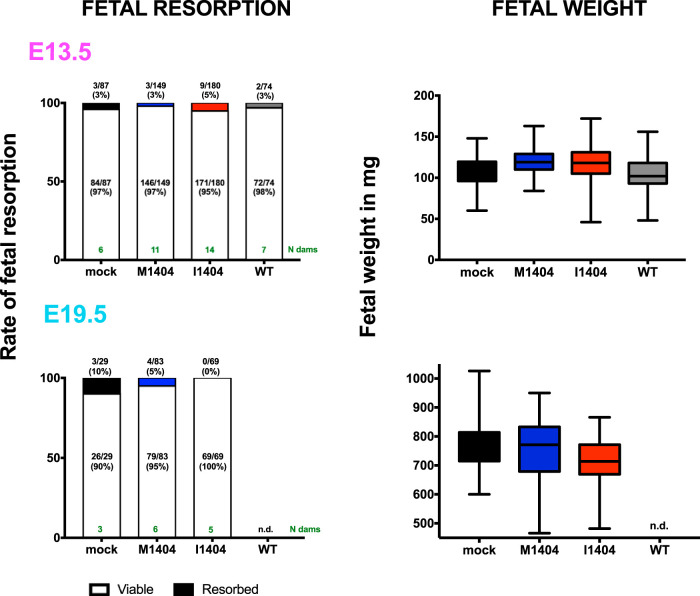
Relative to ZIKV mutant M1404 or the WT, the ZIKV mutant I1404 does not augment fetal death or decrease fetal weight in ZIKV-infected pregnant CD-1 mice. Mice were inoculated as shown in Fig. 4A. No significant differences in the rates of fetal resorption or weight on the gestation day of harvest, E13.5 or E19.5, were detected across groups of pregnant mice that were infected with ZIKV M1404, ZIKV I1404, or the WT or that were mock inoculated. The lines in the middle of each box for the panels on the right show the mean, and error bars show standard deviations. Resorption rates were compared by the use of chi-square statistics. Mean weights were compared by the use of ANOVA multiple-comparison statistics. n.d., not done. The number of dams in each group is shown in green.

To assess whether ZIKV I1404 provides a fitness benefit in pregnancy, we inoculated pregnant CD-1 mice intraperitoneally on gestation day 6.5 (E6.5), similar to the conditions used in an established model (53), with ZIKV WT, ZIKV M1404, ZIKV I1404, or diluent, and compared ZIKV RNA levels in maternal and fetal tissues and fetal weights and resorption rates. Dams were euthanized on gestation day 13.5 (E13.5; full term in mice is E21) in experiment 1 and on E13.5 or E19.5 in experiment 2 (Fig. 4A). Back titration of residual inocula (Fig. 4B) showed that mice were administered similar levels of ZIKV M1404 and ZIKV I1404 RNA in both experiments. The WT inoculum (experiment 1 only) was lower. No statistically significant differences in the rates of fetal resorption or fetal weight were observed in either experiment or on either gestation day (*P* > 0.05, Fisher’s exact test for resorption rates; *P* > 0.05 for mean weights compared with ANOVA multiple comparisons) (Fig. 5). On E13.5, mean maternal spleen ZIKV RNA levels for the WT-inoculated mice were significantly higher than those for ZIKV M1404-inoculated mice (*P* = 0.02, one-way ANOVA) (Fig. 4C), but no significant differences in the rates of ZIKV detection or mean RNA levels in the placentas were observed across the 3 ZIKV-inoculated groups (*P* > 0.05, one-way ANOVA) (Fig. 4D). Despite similar ZIKV RNA levels in the placentas, ZIKV RNA was detected only in fetuses in the ZIKV I1404-inoculated group (9/43 fetuses [20%] for the I1404-inoculated group versus 0/43 fetuses [0%] for the ZIKV M1404-inoculated group; *P* = 0.003, Fisher’s exact test) (Fig. 4E). These fetuses were from 7 different mothers. Sanger sequencing from 3 ZIKV RNA-positive fetuses from different mothers in the ZIKV I1404-inoculated group at E13.5 showed the retention of the I1404 sequence in 1 fetus and reversion to the M1404 sequence in the other 2; for one mother/fetus pair, the reversion was detected only in the fetus and not in the maternal spleen or placenta (Fig. 4F). At E19.5, the magnitude of mean ZIKV RNA levels in spleens and the rates and magnitude of mean ZIKV RNA levels in placentas were significantly higher in mice infected with the I1404 clone than in mice infected with the M1404 clone (*P* < 0.01 for levels in spleen and *P* < 0.001 for levels in placenta, as determined by one-way ANOVA; placenta infection rates, 10/20 [50%] mice infected with the I1404 clone versus 2/20 [10%] mice infected with the M1404 clone [*P* = 0.01, Fisher’s exact test]) (Fig. 4G and H). On E19.5, only 1 fetus head and no fetal bodies in the ZIKV I1404-infected group tested ZIKV RNA positive (Fig. 4I and J) (the rates were not significantly different by Fisher’s exact test). These data show that I1404 confers a fitness advantage in pregnancy in fetuses at E13.5 by increasing the magnitude and rate of fetoplacental infection during murine gestation. Reversion to M1404 as well as the absence of fetal infection by the I1404 clone at E19.5 indicates that the fitness advantages conferred by the mutant may be time and tissue specific and that the wild-type amino acid is favored in some maternal or fetal environments.

### The ZIKV I1404 mutant is more poorly transmitted than ZIKV M1404 by Aedes aegypti.

**FIG 6:**
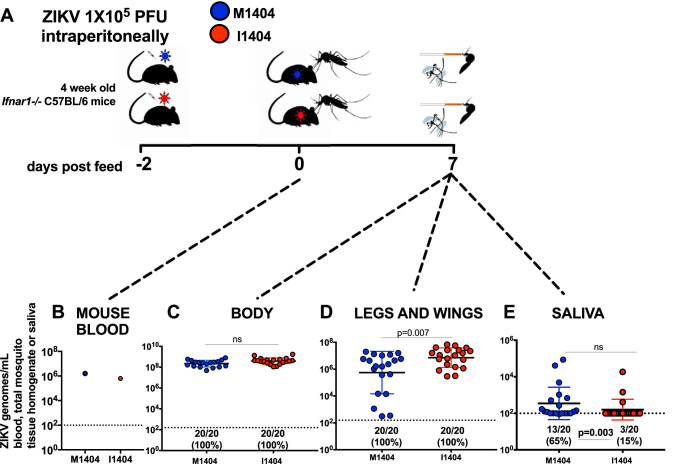
Aedes aegypti mosquitoes transmit ZIKV I1404 less efficiently than they transmit ZIKV M1404. (A) Experimental design showing intraperitoneal inoculation of 1 × 10^5^ PFU per *Ifnar^−/−^* mouse with ZIKV M1404 (blue) or ZIKV I1404 (red) 2 days prior to mosquito feeding, followed by presentation of viremic mice to mosquitoes, a 7-day incubation period, and harvesting of mosquito bodies, legs and wings, and saliva to assess infection, dissemination, and transmission rates and the magnitudes of ZIKV RNA, which were quantified by qRT-PCR. (B) Analysis of mouse blood immediately postfeeding shows that mosquitoes in both groups were exposed to similar quantities of viral RNA. (C to E) ZIKV I1404 infects A. aegypti mosquito bodies (C) and disseminates into legs and wings (D) at rates similar to those for ZIKV M1404 and has significantly higher mean ZIKV RNA levels than ZIKV M1404 but is significantly less transmissible in saliva (E), although the doses transmitted are not different. Each dot represents the mean number of ZIKV genomes measured in mouse blood or individual A. aegypti mosquito tissue or saliva samples at 7 days postfeeding. Only the results for samples with 3/3 replicates with a detectable qRT-PCR value above the limit of detection are reported. The dotted line represents the limit of detection, 2 × 10^2^ ZIKV genome copies/mosquito tissue or blood sample. *P* values comparing mean genome levels are from unpaired *t* tests. Rates were compared by the use of Fisher’s exact statistics.

Given that arbovirus mutations that arise in one host must necessarily be maintained in the alternate host to persist via arthropod-borne cycling in nature, we next considered whether ZIKV I1404 affects transmission by the primary vector, A. aegypti mosquitoes. Mosquitoes were presented to viremic *Ifnar^−/−^* mice infected with ZIKV M1404 or ZIKV I1404 (Fig. 6A). The identity of the sequence at position 1404 in mouse blood was verified by Sanger sequencing. Engorged mosquitoes were held for 7 days after ingesting blood from mice that had matched ZIKV RNA levels (Fig. 6B) and then dissected and assayed to measure the rates and levels of ZIKV RNA in bodies (for infection), legs and wings (disseminated infection), and saliva (a proxy for transmission). The 7-day incubation period was chosen since it represents the time at which mosquitoes exposed to different ZIKV strains beyond the one used here exhibit maximal infection rates (54). All A. aegypti mosquitoes that ingested viremic mouse blood became infected, and the mean ZIKV RNA levels between groups of mosquito bodies were not significantly different (*P* > 0.05, unpaired *t* test) (Fig. 6C). Although all mosquitoes also developed disseminated infections in their legs and wings, the mean ZIKV RNA level was significantly higher for the ZIKV I1404-infected cohort than for the ZIKV M1404-infected cohort (*P* < 0.007, unpaired *t* test) (Fig. 6D). Despite higher dissemination titers, the A. aegypti mosquitoes transmitted ZIKV I1404 more poorly than ZIKV M1404 (3/20 [15%] for ZIKV I1404 versus 13/20 [65%] for ZIKV M1404; *P* = 0.003, Fisher’s exact test) (Fig. 6E). Sanger sequencing of ZIKV from the saliva samples of the mosquitoes in each of the three groups showed retention of the amino acid at 1404 (data not shown). These mosquito data revealed that ZIKV I1404 is less transmissible than ZIKV M1404 in the primary vector at 7 days postfeeding.

## DISCUSSION

Understanding the factors that influence disease and transmissibility can lead to approaches to control ZIKV. We identified a ZIKV mutation, M1404I, that arose *de novo* or that increased in frequency in experimentally inoculated pregnant rhesus macaques. Repeated detection of the mutation in the tissues of multiple pregnant rhesus macaques at a frequency higher than in the inoculum suggests that it confers a selective advantage intrahost in pregnancy. Our experiments in mice confirmed that I1404 increased ZIKV fitness in pregnancy by augmenting the magnitude and the rate of placental infection in mice harvested at E19.5 and by conferring fetal infection in mice harvested at E13.5 in pregnant CD-1 mice inoculated intraperitoneally in the first trimester. In contrast, our studies in nonpregnant rhesus macaques showed that the I1404 clone was less fit than the M1404 clone, producing significantly lower viremias and a decreased frequency in tissues starting from an equal ratio via mixed inoculation, and that the two clones were not different in their capacity to infect fetuses in mice harvested at E19.5. Although similar infectious titers and RNA levels of both competitors were inoculated into nonpregnant macaques, the RNA/PFU ratios measured from Vero cell growth curves indicated that a lower proportion of ZIKV I1404 RNAs than ZIKV M1404 RNAs in the cell monoculture were infectious. The observation of an inferior fitness of ZIKV I1404 compared to ZIKV M1404 in nonpregnant rhesus macaques parallels its low frequency in nonpregnant humans, where only 5 of 543 (<1%) publicly available ZIKV consensus genomes as of August 2020 (https://nextstrain.org/zika?c=gt-NS2B_32) possess the I1404 allele (1 of the 5 was from a microcephalic fetus). We also observed that ZIKV I1404 is not as efficiently transmitted by A. aegypti mosquitoes 7 days after ingesting virus in its first blood feed, which may further explain its low frequency in recent ZIKV outbreaks, where the most common transmission route was human-mosquito-human. Additional studies collecting mosquito saliva at earlier or later times could clarify whether ZIKV I1404 is more transmissible than ZIKV M1404 over many days.

The kinetics of and mechanisms by which I1404 increases ZIKV infection of placental (E19.5) and fetal (E13.5) tissues in murine pregnancy merit further study. Although I1404 enhanced fetoplacental infection, reversion to the wild-type sequence, M1404, in some infected fetuses on gestation day 13.5 suggests that M is the preferred amino acid in some tissues. An alternate possibility is that the ZIKV I1404 stock may have contained M1404 at a frequency lower than the detection limit (ca. 10%) of Sanger sequencing and that ZIKV M1404 increased in frequency to become the consensus in the ZIKV I1404-infected fetuses in which reversion was detected. Detection of ZIKV RNA in 20% of ZIKV I1404-infected fetuses at E13.5 but only 5% of fetal heads and no fetal bodies on E19.5 also raises new questions. The disparity in fetal infection rate between E13.5 and E19.5 is likely not related to the pregnant mice deriving from different cohorts. No intracohort differences in fetal infection rates were observed for the 2 replicate experiments in which dams were inoculated on E13.5. A clearance or reduction of fetal infection below the limit of detection of our quantitative reverse transcription-PCR (qRT-PCR) assays between E13.5 and E19.5 is a possibility. Defining the kinetics of fetal or placental ZIKV RNA levels over time is difficult to assess experimentally since evaluating infection involves destroying the fetus or placenta; even with those limitations, the sacrifice of infected mice earlier in gestation could help further examine whether the fitness advantage exerted by I1404 is accelerated infection kinetics and tropism. It is also possible that ZIKV I1404 infects fetuses at low rates, with the variable rate from experiment 1 to experiment 2 representing a stochastic effect. ZIKV infects many placental cell types, including trophoblasts, endothelial cells, fibroblasts, and fetal macrophages (55, 56), as well as multiple additional fetal cell types (25, 56–59). The I1404 mutation may also confer infection of certain of these cell targets or augment escape from their antiviral responses in ways that M1404 cannot. Together, the results of these studies suggest that the fitness of ZIKV I1404 dynamics may be time, tissue, and host specific, which necessitates caution when extrapolating between mice, nonhuman primates, and humans.

The 1404 locus is in the NS2B-coding region, which encodes a 130-amino-acid protein that acts as a cofactor for NS3, the protease. Relative to other flaviviral proteins, the function(s) of NS2B is poorly understood. NS2B consists of 3 transmembrane domains (TMD) (60, 61). Substitutions in the NS2B TMD decrease yellow fever virus replication (62) and can modify the assembly of Japanese encephalitis virus (60). The 1404 mutation identified in this study is located within NS2B TMD pass 2. Nuclear magnetic resonance of dengue virus NS2B indicates that TMD-TMD interactions might promote membrane fluidity or facilitate interactions with other flavivirus proteins (61). Future studies could focus on subcellular changes in virus-virus or virus-cell interactions mediated by M1404I that may impact cell tropism, infectivity, and immune responses.

**FIG 7:**
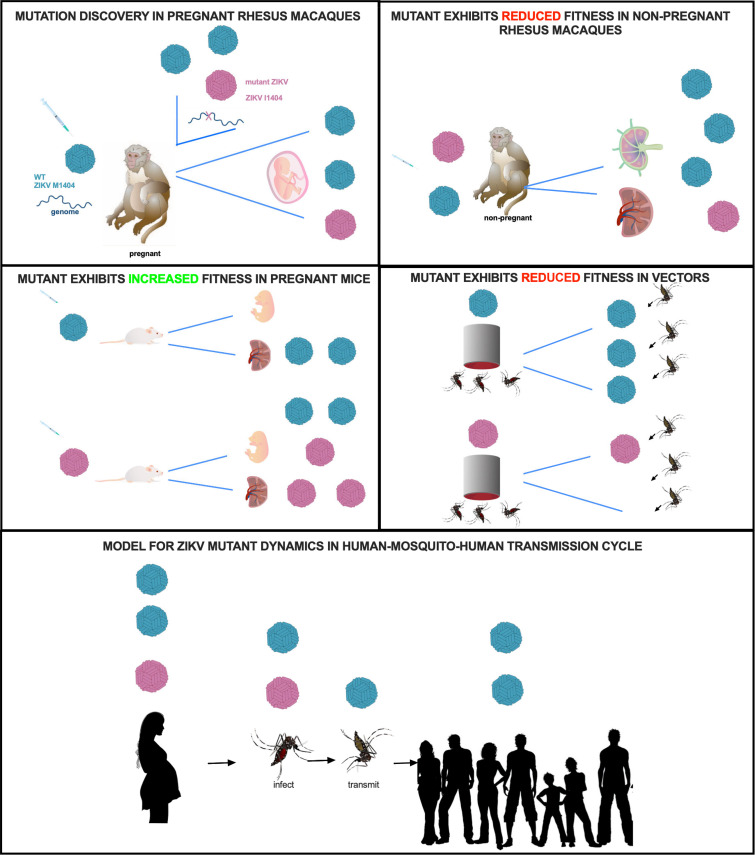
Fitness dynamics for mutant ZIKV. Visual representation of ZIKV M1404 (blue) and ZIKV I1404 (red) fitness dynamics in the experimental systems used in this study. The bottom panel shows a model for the possible transmission dynamics of the mutant in human-mosquito-human cycling, where human infection dynamics are predicted from observations in rhesus macaques.

Here we employed experimental infection of nonpregnant macaques, pregnant mice, and vector mosquitoes to study the fitness of a ZIKV mutation that we initially identified in pregnant rhesus macaques (Fig. 7). Although the mutation studied here does not occur frequently in febrile humans and has been detected in only 1 microcephaly case, these experiments allowed examination of mutation-fitness dynamics in multiple systems. The data from this study support the idea that viruses do not necessarily evolve to become more infectious or virulent, especially if those traits reduce transmissibility. Despite an increase in the frequency of the I1404 mutant in pregnant macaques and even though the I1404 mutation confers increased placental and fetal infection in mice at selected times in gestation, our data show that the I1404 mutant identified in pregnant rhesus macaques is less transmissible by vectors, as measured by lower ZIKV RNA transmission rates in saliva capture assays, and is also less capable of generating levels of viremia in nonpregnant rhesus macaques sufficient to infect feeding vectors. Although higher levels or longer periods of viremia in pregnant macaques may result from fetoplacental spill back to the mother, a pattern that we anecdotally observed in viremic pregnant rhesus macaques whose fetuses died and were removed via fetectomy and that then became aviremic several days later, fetal infection is generally considered a transmission dead end for arboviruses. As such, arboviral mutations that augment fetal infection also need to be neutral or fitness enhancing in mosquitoes to persist in human-mosquito-human cycling. Since we observed decreased fitness, manifest as reduced transmission by vectors at 7 days postfeeding, the M1404I substitution identified in this study is not likely to spread by human-mosquito-human transmission in ZIKV outbreaks. A detailed examination of the incubation period of the I1404 mutant in mosquitoes in laboratory experiments as well as sequencing of ZIKV-infected mosquitoes in the wild could confirm this assertion. A unique feature of this study was the use of two vertebrate models of ZIKV disease as well as vector competence assays. The combined data from these three systems underscore the importance of investigating the consequences of arboviral mutation in both vertebrates and invertebrates, to fully understand their roles in outbreak spread (63).

## MATERIALS AND METHODS

### Rhesus macaques.

Details for the studies with pregnant rhesus macaques are described elsewhere (25, 35). For nonpregnant animals, healthy male or female rhesus macaques (Macaca mulatta) were used in this study. All rhesus macaques were born at the California National Primate Research Center (CNPRC). Animals 5123, 5779, 5606, and 5730 received an HIV envelope protein as part of another study but were never challenged with HIV. Prior to ZIKV inoculation, the animals were housed indoors in stainless steel cages and exposed to a 12-h light/12-h dark cycle at 18 to 23°C with 30 to 70% room humidity. The rhesus macaques were provided water *ad libitum* and received a high-protein commercial chow and fresh fruit supplements. The macaques were observed at least twice daily for clinical signs of disease, including inappetence, stool quality, dehydration, diarrhea, and lethargy, and were given supportive care (including nutritional supplements) as needed. Clinical signs were rare and mild.

### Mice.

Timed pregnant CD-1 mice were purchased from Charles River Laboratories (Sacramento, CA). Animals were housed in a biosafety level 3 (BSL-3) facility at the University of California, Davis, prior to any procedure. A maximum of 4 dams were caged together at each time with a 12-h light/12-h dark cycle at 18 to 23°C with 30 to 70% room humidity, social enhancers, and access to mouse chow and water *ad libitum*. Nonpregnant 2-month-old female Ifnar1 mice (alpha/beta interferon receptor negative C57BL/6, B6.129S2-Ifnar1tm1Agt/Mmjax mice; The Jackson Laboratory, Sacramento, CA) were used as blood meal sources for vector competence studies. Mice were anesthetized prior to mosquito exposure with a mixture of ketamine (75 mg/kg of body weight; VETone Zetamine CIII; Western Medical Supply, Arcadia, CA), xylazine (10 mg/kg; AnaSed; Western Medical Supply, Arcadia, CA), and acepromazine (AceproJect; 1 mg/kg; Western Medical Supply, Arcadia, CA) solution administered intraperitoneally. Immediately after the mosquito feeds, the mice were euthanized, while they were still under anesthesia via exsanguination, using cardiac puncture followed by cervical dislocation.

### Animal use.

The University of California, Davis, is accredited by the Association for Assessment and Accreditation Laboratory Animal Care International (AAALAC). Animal care was performed in compliance with the 2011 *Guide for the Care and Use of Laboratory Animals* provided by the Institute for Laboratory Animal Research (64), and both the rhesus macaque and mouse studies were approved by the Institutional Animal Care and Use Committee (IACUC) of the University of California, Davis. All mouse procedures were approved under protocol 19404. All rhesus macaque procedures were approved under protocols 19211 and 19695.

### Mosquitoes.

Mosquitoes of the fourth generation of Aedes aegypti originally collected in 2015 in Puerto Rico were used in this study. The mosquitoes were maintained in a colony in an insectary at the University of California, Davis. The mosquitoes were provided 10% sucrose (Thermo Fisher Scientific, Emeryville, CA) prior to presentation to mice. At 24 h prior to exposure to a mouse, the mosquitoes were transferred into pint cartons and transported into a BSL-3 facility to acclimate in a humidified chamber set to a 12-h light/12-h dark cycle at 27°C with 80% humidity. After the ingestion of blood from viremic ZIKV-infected mice, the mosquitoes were presented with 10% sucrose *ad libitum*.

### Cell lines.

African green monkey kidney (Vero) cells (ATCC CCL-81; ATCC, Manassas, VA) were cultured at 37°C in 5% CO_2_ in Dulbecco’s modified Eagle medium (DMEM; Gibco, Thermo Fisher Scientific, Emeryville, CA) supplemented with 2% fetal bovine serum (FBS; Gibco, Thermo Fisher Scientific, Emeryville, CA) and 1% penicillin-streptomycin (Gibco, Thermo Fisher Scientific, Emeryville, CA). Baby hamster kidney (BHK21) cells (ATCC CCL-10; ATCC, Manassas, VA) were cultured under the same conditions as the Vero cells but were supplemented with 10% FBS. Aedes albopictus (C6/36) cells (ATCC CRL-1660; ATCC, Manassas, VA) were cultured in Schneider’s insect medium (Caisson Labs, Smithfield, UT) supplemented with 20% FBS and 1% penicillin-streptomycin at 28°C under atmospheric CO_2_.

### Viruses.

**(i) Wild-type Zika virus stock.** For growth curves and *in vivo* experiments in pregnant and nonpregnant rhesus macaques and pregnant mice, the 2015 Brazilian ZIKV strain SPH2015 (GenBank accession number KU321639) was used. This virus was originally isolated from a human blood transfusion recipient in São Paulo, Brazil, and then passaged 3 times in Vero cells. We refer to this strain as the wild type (WT) throughout the paper. A 2015 Puerto Rico ZIKV strain, PRVABC59 (GenBank accession number KU501215.1, Vero cell passage 4), was also used in pregnant rhesus macaques whose ZIKV isolates were sequenced for this project. We refer to this strain as ZIKV Puerto Rico 2015 throughout the paper.

**(ii) Generation of infectious clone-derived M1404 and I1404 Zika viruses.** To focus on the 1404 locus as a determinant of phenotype, we generated 2 infectious clones that were identical in sequence except for the amino acid at position 1404. To start, we modified a ZIKV infectious clone made from the sequence of a 2015 Brazilian ZIKV strain, Paraiba_01/2015 (17), to match the amino acid sequence of the WT strain (SPH2015), which encodes M1404. Six mutations were inserted into the Pariaba_01/2015 clone at positions V313I, Y916H, V1143M, H1857Y, I2295M, and I2445M of the ZIKV polyprotein to generate the WT amino acid sequence of strain SPH2015. We refer to this clone as ZIKV M1404. Next, the M1404 clone was mutated to change the amino acid at position 1404 from G > A (AUG [methionine] > AUA [isoleucine]) to generate the I1404 clone. The ZIKV I1404 clone is identical to the M1404 clone, except at NS2B locus 1404. Each of the mutagenized loci were verified by Sanger sequencing. Infectious viruses were rescued from the ZIKV M1404 and ZIKV I1404 clones by electroporating plasmid DNA into BHK cells. Plasmid DNA (2.5 μg) was electroporated at 110 V, a 1,750-μF capacitance, and no resistance using an ECM 630 electro-cell manipulator (BTX Harvard Apparatus, Holliston, MA) into T75 flasks of BHK21 cells at ≈50% confluence. The cells were then centrifuged for 5 min at 1,500 rpm and resuspended in DMEM in T25 flasks for 3 days of incubation at 37°C with 5% CO_2_. After 3 days, the supernatant was collected, spun at 1,500 rpm for 5 min to remove the cell debris, and stored at −80°C in 400-μl aliquots. These recovered infectious clone-derived viruses, termed ZIKV M1404 and ZIKV I1404, were used in growth curve, mosquito, pregnant mouse, and nonpregnant rhesus macaque experiments. The genotypic integrity of both infectious clone-derived viruses was verified by whole-genome Sanger sequencing from the electroporation-harvested stocks.

### Zika virus titrations using plaque assays.

Infectious ZIKVs from electroporation-rescued stocks of infectious clone-derived viruses, inocula, growth curves, mosquito saliva, and qRT-PCR-positive mouse fetuses were titrated using plaque assays. Plaque assays were performed in confluent 6-well plates of Vero cells that were inoculated with 250 μl of 10-fold dilutions of virus stock or sample resuspended in 2% FBS–DMEM. Cells were incubated for 1 h at 37°C in 5% CO_2_, and the plates were rocked every 15 min to prevent cell death due to desiccation. After 1 h, 3 ml of 0.5% agarose (Thermo Fisher Scientific, Emeryville, CA) mixed with 2% FBS–DMEM was added to each well to generate a solid agar plug. The cells were then incubated for 7 days at 37°C in 5% CO_2._ After 7 days, the cells were fixed with 4% formalin (Thermo Fisher Scientific, Emeryville, CA) for 30 min, the agar plugs were removed, and the cells were stained with 0.025% crystal violet (Thermo Fisher Scientific, Emeryville, CA) in 20% ethanol (Thermo Fisher Scientific, Emeryville, CA) in order to visualize and quantify the plaques. Samples were tested in duplicate, and the average titer is reported as the number of plaques visible against a white background. The limit of detection was 40 PFU.

### Zika virus RNA isolation.

ZIKV RNA was isolated using a MagMAX viral RNA extraction kit (Thermo Fisher Scientific, Emeryville, CA) or the QIAzol reagent (Qiagen, Redwood City, CA). The MagMAX system was used to extract ZIKV RNA from growth curve supernatants, rhesus macaque plasma, and homogenized mosquito bodies, legs and wings, and saliva. The MagMAX viral RNA extraction kit was used according to the manufacturer’s recommendations. For cell supernatant and mosquito homogenates, a total of 100 μl of sample was extracted, and for rhesus macaque plasma, the volume extracted was 300 μl. Maternal spleens, placentas, and fetuses from the mouse experiments and solid tissues from the rhesus macaque were extracted using the QIAzol reagent. Tissues stored in RNAlater solution (Sigma-Aldrich, St. Louis, MO) were first removed from that solution prior to RNA extraction. Using clean forceps and scissors, tissues were removed from the RNAlater solution, and a portion of between 20 and 50 mg was cut and placed in a preweighed tube containing a 0.5-mm glass ball bearing (Thermo Fisher Scientific, Emeryville, CA). The tubes were then reweighed and 900 μl of QIAzol solution was added, before trituration in a TissueLyser (Retsch, Haan, Germany) machine. The tissues were homogenized for 2 m at 30 shakes/s. If liquefaction of the tissues was incomplete, the samples were homogenized for an additional 2 m at 30 shakes/s. The homogenate was centrifuged for 2 min at 14,000 × *g* to clarify the supernatant, which was tested. Viral RNA was extracted from the homogenates following the manufacturer's instructions provided in the kit. The QIAzol protocol was modified for the E19.5 fetuses since they were large. E19.5 fetus samples were homogenized in 1 ml of DMEM, and 200 μl of the homogenate was added to 900 μl of lysis reagent, after which the protocol followed the manufacturer's instructions. All RNA extracts were eluted in 60 μl of Qiagen elution buffer and archived at −80°C until further analysis.

### Zika virus RNA quantification by qRT-PCR.

ZIKV RNA samples were each measured in triplicate on an Applied Biosystems ViiA 7 machine using a TaqMan Fast virus 1-step master mix (Thermo Fisher Scientific, Emeryville, CA) with primers ZIKV 1087 forward (CCGCTGCCCAACACAAG) and ZIKV 1163c reverse (CCACTAACGTTCTTTTGCAGACAT) and the probe ZIKV 1108-FAM (AGCCTACCTTGACAAGCAGTCAGACACTCAA), where FAM indicates 6-carboxyfluorescein, according to the protocol described by Lanciotti et al. (65). The protocol was modified by increasing the initial volume of the sample tested to 9.6 μl to increase sensitivity. Samples were considered positive only if all three replicates yielded a detectable cycle threshold (*C_T_*) value of less than the cutoff of the assay, which was 40. For each 96-well plate in which samples were tested, a standard curve was generated from serial dilutions of a synthetic DNA of known concentration corresponding to the qRT-PCR target region. The reported limit of detection (LOD) on each graph shows the mean for all samples with a detectable *C_T_* that were included in the graph. Where means are reported for a group of measurements, samples with no detectable ZIKV RNA were included in measurements where their values were reported at the LOD.

### *In vitro* Zika virus growth assays.

Vero and C6/36 cells were inoculated in triplicate with ZIKV M1404, ZIKV I1404, or the WT at a multiplicity of infection (MOI) of 0.01 PFU/cell (Vero cells only) or 0.5 PFU/cell. Cells in one well in the tissue culture plates (Thermo Fisher Scientific, Emeryville, CA) were counted immediately prior to infection, and the MOI was adjusted according to the cell count by diluting each virus stock based on its predetermined Vero cell plaque assay titer. The cells were inoculated by overlaying 150 μl of virus for 1 h at 37°C in 5% CO_2_ for Vero cells and 28°C in ambient CO_2_ for C6/36 cells, with gentle rocking every 15 min. After 1 h, the cells were washed three times with phosphate-buffered saline (PBS; Thermo Fisher Scientific, Emeryville, CA) to remove residual unbound viruses, and 2 ml/well of DMEM was added. At time points of 0, 12, 24, 48, and 72 h postinoculation (hpi) for C6/36 cells and Vero cells as well as 96 hpi for Vero cells, 160 μl/well was collected and archived at −80°C until the cells were tested in triplicate to determine ZIKV RNA levels by qRT-PCR. The data shown are the percentage of the mean residual input of ZIKV RNA (in number of genomes per milliliter) at 0 hpi. Each data point shows the mean measurement for 3 wells that were each measured in triplicate by qRT-PCR.

### Experimental inoculation of nonpregnant rhesus macaques with Zika virus.

Studies with pregnant rhesus macaques were described elsewhere (23, 33). Nonpregnant female and male rhesus macaques were inoculated subcutaneously or intravenously with 1 ml of the ZIKV WT at 1 × 10^4^ PFU; a 1:1 mixture of ZIKV M1404 and ZIKV I1404, in which 1 × 10^3^ PFU of each virus, verified by titration prior to mixing, was in the inoculum; or 2 × 10^3^ PFU of ZIKV I1404. All inocula were back titrated immediately after inoculation without freezing using plaque assays to verify the administered doses. All inocula were resequenced to verify the identity at the 1404 locus. The mixed inoculum was also checked prior to inoculation by next-generation sequencing of the sequence flanking the 1404 locus and was verified to contain 49% ZIKV isolates with M1404 and 51% ZIKV isolates with I1404. Urine and blood were collected from the rhesus macaques daily for 7 days and then every other day until 14 dpi. The macaques were anesthetized with ketamine hydrochloride (10 mg/kg; Western Medical Supply, Arcadia, CA), and the samples were processed according to previously described methodologies (35). At 14 dpi, the rhesus macaques were euthanized and necropsied. During the necropsies, tissues were grossly evaluated *in situ*, then excised using forceps, and then dissected with disposable razor blades to minimize cross-contamination. Tissues were either snap-frozen by immersion in liquid nitrogen or stored in RNAlater solution. Samples were held in the RNAlater solution at 4°C for 24 h and then transferred to −80°C for further analysis.

### Experimental inoculation of pregnant mice with Zika virus.

Two ZIKV experiments were performed with pregnant mice. The numbers of mice are shown in the appropriate figures. For both experiments, the mice were sedated with isoflurane and inoculated intraperitoneally with 100 μl of 1 × 10^5^ PFU of the ZIKV WT, ZIKV M1404, or ZIKV I1404 or DMEM on gestation day 6.5 (E6.5). The inoculum doses were verified by qRT-PCR. All mice in experiment 1 and some mice in experiment 2 were euthanized on E13.5 (full term is 21 days). Some mice in experiment 2 were euthanized later, at E19.5. After inoculation, the mice were monitored twice daily by visual observation. On E13.5 or E19.5, the mice were sedated with isoflurane and euthanized by cervical dislocation. The uterine horns were exposed and visually observed for viable or aborted/resorbed fetuses. The maternal spleen was excised and cut in half. Half was placed in a 2-ml tube containing 1 ml of RNAlater solution, and the other half was stored in a 2-ml tube (Thermo Fisher Scientific, Emeryville, CA) with 1 ml of DMEM. The placentas were collected in 2-ml tubes containing 1 ml of DMEM. The fetuses were weighed and their length was measured, and then they were collected in a 2-ml tube containing 1 ml of DMEM. Between work with each dam, the forceps and scissors were immersed in 10% bleach and then 70% ethanol solution to minimize cross-contamination across animals. Samples in DMEM were immediately stored at −80°C. Samples were stored in RNAlater solution at 4°C for 24 h and then transferred to −80°C until further analysis.

### Experimental vector competence of Zika virus in Aedes aegypti.

Ifnar1^−/−^ C57BL/6 mice were intraperitoneally inoculated with 1 × 10^5^ PFU of ZIKV WT, ZIKV M1404, or ZIKV I1404 2 days prior to presentation to female A. aegypti mosquitoes. Prior to mosquito presentation, the mice were anesthetized with a ketamine (75 mg/kg; Western Medical Supply, Arcadia, CA), xylazine (10 mg/kg; Western Medical Supply, Arcadia, CA), and acepromazine (1 mg/kg; Western Medical Supply, Arcadia, CA) solution, administered intraperitoneally. Viremic mice were presented to sugar-deprived mosquitoes for 1 h. After 1 h, mouse blood was collected immediately after mosquito feeding to measure ZIKV RNA levels and to verify the identity of the sequence at position 1404 by Sanger sequencing. The mice were then euthanized by cervical dislocation. Engorged female mosquitoes were sorted from nonfed individuals by visual examination and then held for 7 days. On day 7, the mosquitoes were cold anesthetized for 3 to 5 min at −20°C, and then their legs and wings were removed with forceps while they were immobilized on ice. Saliva was collected by inserting the proboscis into a glass capillary tube (Thermo Fisher Scientific, Emeryville, CA) containing FBS (Thermo Fisher Scientific, Emeryville, CA) for 30 min. Mosquito bodies, legs and wings, and saliva, including the capillary, were placed into 2-ml tubes containing 250 μl of DMEM and a glass bead (Thermo Fisher Scientific, Emeryville, CA). The samples were immediately archived at −80°C for further analysis. After thawing, mosquito samples were homogenized for 2 min at 30 shakes/s in a TissueLyser apparatus. The homogenate was centrifuged for 2 min at 14,000 × *g* to clarify the supernatant, which was tested to measure viral RNA levels and, for selected samples, to identify the sequence at position 1404 by Sanger sequencing.

### Zika virus sequencing and sequence analyses.

The plasmid sequences of infectious clones and the identities of virus stocks as well as selected samples from mice and mosquitoes were verified by Sanger sequencing, using primers flanking the entire genome or position 1404 (see Table S1 in the supplemental material). Extracted RNA samples were amplified using a Qiagen (Redwood City, CA) one-step reverse transcription-PCR (RT-PCR) kit, forward primer AGCTGTTGGCCTGATATGCG, and reverse primer AGCTGCAAAGGGTATGGCTA. The cycling conditions were as follows: 50°C for 30 min, 95°C for 15 min, and 40 cycles of 94°C for 1 min, 57°C for 1 min, and 72°C for 1 min, followed by 72°C for 10 min and a hold at 4°C. Samples were sequenced at the University of California, Davis, Sequencing Core Facility. Chromatograms were visualized and sequences were called using the Sequencher program (Gene Codes, Ann Arbor, MI). The complete ZIKV genome from the inoculum and samples from pregnant rhesus macaque 5338, whose fetus died at 7 dpi, was sequenced at both the University of California, San Francisco, using an established protocol (66), and Lawrence Livermore National Laboratories, using a different established protocol (67). Samples from all other rhesus macaques and other samples in this study were sequenced using a different next-generation sequencing protocol that was previously described (68, 69) and adapted here to flank the sequence from position 1404. After viral RNA isolation using QIAzol solution and RNA quantification by qRT-PCR, at least 1,000 genome copies/sample were used to generate libraries for sequencing. Five microliters of ZIKV RNA was used in a cDNA synthesis reaction with a SuperScript IV (SSIV) kit (Invitrogen, Thermo Fisher Scientific, Emeryville, CA), in addition to 6 μl of nuclease-free water, 1 μl of a 10 mM deoxynucleoside triphosphate (dNTP) mix, and 1 μl of random hexamers. The mixture was heated to 70°C for 7 min and immediately placed on ice. A new mixture containing 4 μl of 5× SSIV buffer with 1 μl of 100 mM dithiothreitol, 1 μl of RNase inhibitor, and 1 μl of SSIV reverse transcriptase was added, and cDNA synthesis occurred under thermocycler conditions of 23°C for 10 min, 50°C for 45 min, 55°C for 15 min, and 80°C for 10 min and then a hold at 4°C until further use. The sequence at position 1404 was amplified using 1 μl each of 10 μM forward primer CCCTAGCGAAGTACTCACAGCT and reverse primer TACACTCCATCTGTGGTCTCCC, 2.5 μl of cDNA, 15 μl of nuclease-free water, 0.5 μl of Q5 high-fidelity DNA polymerase (New England Biolabs, Ipswich, MA), 1 μl of 10 mM dNTPs, and 5 μl of 5× Q5 reaction buffer, followed by 98°C for 30 s, 95°C for 15 s, and 65°C for 5 min, then repetition of steps 2 and 3 for 34 additional cycles, and then a hold at 4°C until further use. The PCR products were purified using Agencourt AMPure XP magnetic beads (Beckman Coulter, San Jose, CA) at a 1.8:1 ratio of beads to sample. Sequencing libraries were next generated using a Kapa Hyper preparation kit (Roche, Pleasanton, CA). Specifically, the ends were repaired by mixing 1.75 μl of end-repair and A-tailing buffer, 0.75 μl of end-repair A-tailing enzyme mix, and 12.5 μl of amplified DNA, followed by incubation at 20°C for 30 min and then 65°C for 30 min. For adaptor ligations, 2.5 μl of 250 nM NEXTflex dual-index DNA barcodes (Bioo Scientific, Austin, TX) was used with 15 μl of the end-repair reaction product, 2.5 μl of DNA ligase, and 7.5 μl of ligation buffer, which were incubated at 20°C for 15 min. This procedure was followed by a postligation cleanup using Agencourt AMPure XP magnetic beads at a ratio of 0.8:1 beads to sample. The sequencing library was then amplified using 17 μl of 2× Kapa HiFi HotStart ReadyMix (Roche, Pleasanton, CA), 2 μl of Illumina (Redwood City, CA) primer mix, and 15 μl of adaptor-ligated library, followed by 98°C for 45 s, 98°C for 15 s, 60°C for 30 s, and 72°C for 30 s and then a repetition of steps 2 to 4 for 8 cycles, followed by 72°C for 1 min and a hold at 4°C until further use. The amplified samples were then cleaned using Agencourt AMPure XP (Beckman Coulter, San Diego, CA) magnetic beads in a ratio of 0.8:1 beads to sample. DNA library sizes were then analyzed using a Bioanalyzer DNA 1000 kit (Agilent Santa Clara, CA), and the DNA concentration was quantified using a Qubit high-sensitivity DNA kit (Thermo Fisher Scientific, Emeryville, CA). Libraries were diluted to 2 nM in 10 mM Tris-EDTA (TE) buffer, and samples were sequenced with a MiSeq apparatus (Illumina, Hayward, CA), using a paired-end approach. We used a previously described work flow (63) to determine M1404I allele frequencies. Briefly, sequence reads were trimmed to remove the primer sequences and low-quality base calls before they were aligned to the Zika virus WT SPH2015 reference genome using the BWA-mem program (70). Mutants with over a 3% minor allele frequency were called using the SAMtools mpileup tool (71) and were filtered according to frequency and strand biases. After sequencing, the ratios of G (encoding M1404) versus A (encoding I1404) were calculated and are represented as a percentage of the total sequencing depth at the locus.

### Statistical analyses.

Statistical analyses were performed using GraphPad Prism (version 7) software (GraphPad Software, San Diego, CA). The tests used are indicated in Results and the figure legends. Statistical significance is denoted by *P* values of less than 0.05.

### Data availability.

Sequencing data are available in the NCBI SRA at accession number PRJNA556052.

## Supplementary Material

Supplemental file 1
