# Supplementary material for: Two Sides of a Coin: a Zika Virus Mutation Selected in Pregnant Rhesus Macaques Promotes Fetal Infection in Mice but at a Cost of Reduced Fitness in Nonpregnant Macaques and Diminished Transmissibility by Vectors
Source: J Virol. 2020 Nov 23;94(24):e01605-20. doi: 10.1128/JVI.01605-20 (PMC7925200; doi:10.1128/JVI.01605-20)
Supplement: Supplemental file 1 [file JVI.01605-20-s0001.pdf]

SUPPLEMENTAL TABLES

Supplemental Table 1. Primers used for Sanger sequencing ZIKV in this study.

ZIKV Primer Name	Primer Sequence (5' to 3')
28p	CGACAGTTCGAGTTTGAAGCG
430p	CCAGTGTCGGAATTGTTGGC
563n	CCCCAGTGTGGTTGGAAAAG
904p	GGCTTTTGGGAAGCTCAACG
1048n	ACAACATCAACCCAGGTCCC
1200p	CCAACACAAGGTGAAGCCTAC
1489n	GGCGTTATCTCAACCTTCGC
1618p	ACAAGGAGTGGTTCCATGACA
1776n	TGTGAACGGCTCCTTCTTGAC
1970p	GTATGCAGGGACAGATGGACC
2204n	TTTTCCGATGGTGCTGCCAC
2301p	GGCAAGGGCATCCATCAAAT
2537n	ACCGCATCTCGTTTCCTTCTT
2749p	CGGTCGTTGTGGGATCTGTAA
2979n	GAAATACCCCGAACCCGTGA
3246p	CACCACAACACCAGAGAGGG
3393n	TGGTTGATCTCAGGGACGGT
3711p	AAGCTTGCAATTCTGATGGGTG
3894n	CAGTTTGCAGAAGGCACGAG
4144p	CCTTGGGACTAACCGCTGTG
4299n	TATCTGCTTTGGCGAACCCTC
4634p	GCCTGCTCCCAAGGAAGTAAA
4809n	CAAGTCTCCCTTCACCGCTC
5197p	ATCCTGGAGCTGGGAAAACC
5342n	GTAACGCACTGGAAGCCCT
5814p	CAAGAGTGGGACTTCGTCGTA
5924n	GCCATCAAGTATGACCGGCT
6379p	TGCTCAAACCGAGGTGGATG
6516n	TCATGTGTCCTGGCAGTGTT
6878p	CATGGTAGCAGTGGGTCTTCT
7063n	ATGAGAGTTGTCAGAGCGGC
7451p	CATCTCCAGTGCCGTTCTGC
7573n	GCTGTGGAGGAGTTCCAGTAT
7925p	CTGGAGTTACTACGCCGCC
8075n	CGCCATGTGAAAGACGTCCA
8510p	CGTTGAGAGGATCCGCAGTG
8649n	GCCTGACAACCCCGTTTATG

9059p	GGAATTTGGAAAGGCCAAGGG
9206n	TAATCCCAGCCCTTCAACACC
9614p	GCAGAGCAACGGATGGGATA
9763n	GAGGGTTTCCACTCCTGTGT
10220p	TCTTATAGGGCACAGACCACG
10365n	GTGTGGACCCTTCTTCACCC
10803n	CCATGGATTTCCCCACACCG

Supplemental Table 2. Deep sequencing data showing the percentage of reads that encode ZIKV I1404 in inocula and experimentally infected pregnant rhesus macaques. For most samples, a targeted sequencing approach flanking ZIKV polyprotein amino acid 1404 was used. [a],[b] and [c] show data from different laboratories. [a] is The Scripps Research Institute, [b] is University of California San Francisco, and [c] is Lawrence Livermore National Laboratories. PR is Puerto Rico, WT is SPH2015, a Brazilian strain, KU321639, N/A is not applicable, DPI is day post inoculation, IV is intravenous, IA is intraamniotic, SC is subcutaneous.

ANIMAL IDENTIFIER or INOCULUM	ZIKV STRAIN	INOCULATION ROUTE	FETAL OUTCOME	STUDY END POINT (DPI)	TISSUE	DPI	COVERAGE AT 1404	% I1404 MUTANT (AUA)
Inoculum	PR 2015	SC	N/A	N/A	N/A	N/A	2242	0%[a]
Inoculum	WT	IV + IA	N/A	N/A	N/A	N/A	5705	0.2% [b]
Inoculum	WT	IV + IA	N/A	N/A	N/A	N/A	2296	1.3% [a]
5731	PR 2015	SC	Near term	125	Maternal plasma	5	2706	0%[a]
5731	PR 2015	SC	Near term	125	Maternal spleen	125	15	14% [a]
5388	WT	IV + IA	Death	7	Amniotic fluid	2	1175	25%[b]
5388	WT	IV + IA	Death	7	Amniotic fluid	7	2564	17%[a]
5388	WT	IV + IA	Death	7	Gestational Sac	7	8020	14% [a]
5388	WT	IV + IA	Death	7	Amniotic membrane	7	309	25%[c]
5388	WT	IV + IA	Death	7	Amniotic membrane	7	4492	28% [a]
5388	WT	IV + IA	Death	7	Placenta full thickness	7	5477	5% [a]
5388	WT	IV + IA	Death	7	Vagina	7	777	19%[c]
5388	WT	IV + IA	Death	7	Spleen	7	6235	0% [a]
5388	WT	IV + IA	Death	7	Inguinal Lymph node	7	4332	0.1% [a]
5388	WT	IV + IA	Death	7	Fetal Lung	7	6236	0% [a]
5388	WT	IV + IA	Death	7	Fetal Intestine	7	21444	0% [a]
5388	WT	IV + IA	Death	7	Fetal Brain	7	4581	0.2% [a]
5592	WT	IV + IA	Death	14	Amniotic fluid	14	13	8% [a]
5592	WT	IV + IA	Death	14	Inguinal Lymph node	14	2389	0% [a]
5592	WT	IV + IA	Death	14	Amniotic membrane	14	7695	0% [a]
5592	WT	IV + IA	Death	14	Fetal intestine	14	9362	0.1% [a]
5592	WT	IV + IA	Death	14	Fetal kidney	14	6812	0.1% [a]

5592	WT	IV + IA	Death	14	Fetal brain	14	2849	0.1%[a]
4817	WT	IV + IA	Near term	105	Amniotic fluid	2	8000	0%[c]
4817	WT	IV + IA	Near term	105	Amniotic fluid	14	438486	4%[a]
4817	WT	IV + IA	Near term	105	Amniotic fluid	21	2560	5%[a]
4817	WT	IV + IA	Near term	105	Vagina	105	1232	9%[C]
5846	WT	IV + IA	Near term	87	Amniotic fluid	7	15869	13%[a]
5846	WT	IV + IA	Near term	87	Amniotic fluid	14	5439	12%[a]
5846	WT	IV + IA	Near term	87	Amniotic fluid	21	10045	35%[a]
5846	WT	IV + IA	Near term	87	Amniotic fluid	28	333	2%[C]
5846	WT	IV + IA	Near term	87	Amniotic membrane	87	217	7%[C]
5846	WT	IV + IA	Near term	87	Fetal seminal vesicle	87	557	3%[C]
5846	WT	IV + IA	Near term	87	Urine	40	1929	11%[C]
5846	WT	IV + IA	Near term	87	Urine	77	1725	37%[C]
5846	WT	IV + IA	Near term	87	Placenta	87	2991	0%[C]
5846	WT	IV + IA	Near term	87	Fetal brain	87	2800	0%[c]
5846	WT	IV + IA	Near term	87	Amniotic fluid	2	7842	0.3%[a]
5207	WT	IV + IA	Near term	65	Amniotic fluid	2	8797	14%[C]
5207	WT	IV + IA	Near term	65	Placenta	65	5130	4%[C]
5462	WT + PR 2015	IV + IA	Death	73	Amniotic fluid	7	6151	0%[a]
5462	WT	IV + IA	Death	73	Amniotic fluid	14	6686	0.4%[a]
5462	WT	IV + IA	Death	73	Fetal Lung	70	5016	0%[a]
5507	PR 2015	SC	Death	30	Amniotic membrane	30	5052	0%[a]
5512	PR 2015	SC	Near term	121	Amniotic membrane	121	5318	0%[a]
5803	WT + PR 2015	IV + IA	Death	42	Fetal lung	42	14467	0%[a]
5803	WT+ PR 2015	IV + IA	Death	42	Fetal ileum	42	7137	0%[a]
5803	WT+ PR 2015	IV + IA	Death	42	Fetal kidney	42	12886	0%[a]
5803	WT + PR 2015	IV + IA	Death	42	Fetal brain – parietal lobe	42	5491	0.1%[a]
5803	WT + PR 2015	IV + IA	Death	42	Placenta full thickness	42	11923	0%[a]
5803	WT + PR 2015	IV + IA	Death	42	Amniotic membrane	42	4935	0%[a]

Supplemental Table 3. Deep sequencing data showing the percentage of reads at ZIKV 1404 in non-pregnant rhesus macaques. '1:1 Mix' denotes an equal mixture of ZIKV M1404 and I1404. LN is lymph node. DPI is days post-inoculation. SC is subcutaneous. These same data are shown graphically in Figure 2E.

ANIMAL IDENTIFIER or INOCULUM	ZIKV STRAIN	INOCULATION ROUTE	TISSUE	DPI	NUCLEOTIDE COVERAGE AT 1404	% I1404 MUTANT
INOCULUM	1:1 Mix	N/A	N/A	N/A	1013	51%
5123	1:1 Mix	SC	Plasma	3	7920	18%
5123	1:1 Mix	SC	Plasma	5	7	14%
5123	1:1 Mix	SC	Inguinal LN	15	1607	32%
5123	1:1 Mix	SC	Mesenteric LN	15	881	22%
5123	1:1 Mix	SC	Spleen	15	170	25%
5779	1:1 Mix	SC	Plasma	3	7922	27%
5779	1:1 Mix	SC	Plasma	5	7888	21%
5779	1:1 Mix	SC	Ileum	15	384	9%
5779	1:1 Mix	SC	Inguinal LN	15	412	50%
5779	1:1 Mix	SC	Mesenteric LN	15	126	45%
5779	1:1 Mix	SC	Spleen	15	496	31%
